# Letter from the Editor in Chief

**DOI:** 10.19102/icrm.2018.090808

**Published:** 2018-08-15

**Authors:** Moussa Mansour


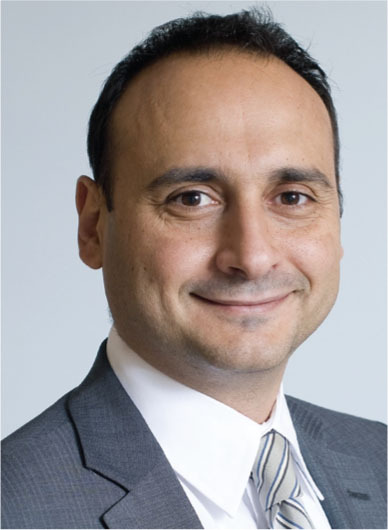


Dear Readers,

Cardiovascular implantable electronic device (CIED) infection is a serious adverse event that can occur following device placement. To date, the number of CIED-related infections has been increasing, with the rate of escalation having already surpassed the rate of growth of cardiac device implantation.^1^ One major reason for this trend is that patients undergoing CIED implantation now are predominantly older and have more comorbidities as compared with those being implanted 10 years ago.

Infected hardware often contains vegetations that can be detected by echocardiogram prior to or during the procedure. Surgical extraction has been the preferable management option in the presence of large vegetations; however, surgical extraction is associated with increased morbidity and cost versus percutaneous extraction.

Vacuum-assisted thrombectomy has been used in the field of interventional cardiology for some time. More recently, the same devices have been introduced to the field of cardiac electrophysiology, with small feasibility studies having demonstrated their value in debulking vegetations prior to or in conjunction with percutaneous lead extraction.^2–4^ In the current issue of *The Journal of Innovations in Cardiac Rhythm Management*, the authors of an original article titled “AngioVac Debulking in Endocarditis Patients with Large, Device-related Vegetations”^5^ describe a series of five cases of vacuum-assisted removal of lead-related vegetations and CIED extraction. The debridement of the vegetations was performed either prior to or along with percutaneous lead extraction and all extractions were successful despite the larger size of the vegetations (ie, up to 3.9 cm).

Vacuum-assisted debulking of infected CIED leads represents an elegant adaption of an existing technology to fulfill an unmet need in cardiac electrophysiology. However, experience using this technology in combination with CIED extraction remains limited. Larger studies are needed to confirm the benefits suggested in the existing literature.

Best wishes for a relaxing rest of the summer and I hope that you enjoy reading this issue of *The Journal of Innovations in Cardiac Rhythm Management.*

Sincerely,


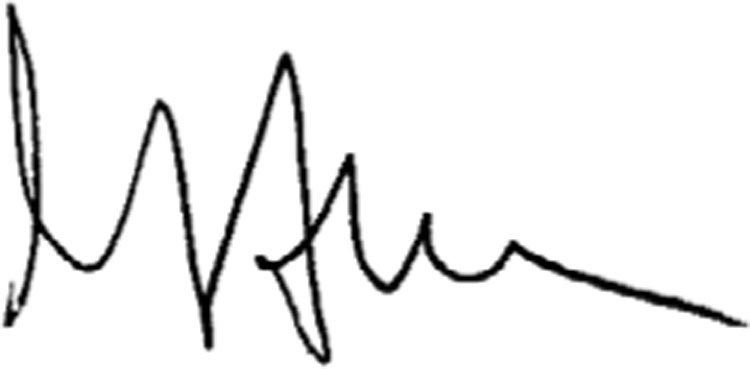


Moussa Mansour, MD, FHRS, FACC

Editor in Chief

The Journal of Innovations in Cardiac Rhythm Management

MMansour@InnovationsInCRM.com

Director, Atrial Fibrillation Program

Jeremy Ruskin and Dan Starks Endowed Chair in Cardiology

Massachusetts General Hospital

Boston, MA 02114
